# Association of Carotid Intima Media Thickness With Metabolic Syndrome Among Low-Income Middle-Aged and Elderly Chinese: A Population-Based Cross-Sectional Study

**DOI:** 10.3389/fcvm.2021.669245

**Published:** 2021-11-19

**Authors:** Qiaoxia Yang, Qiuxing Lin, Dandan Guo, Hanhua Wang, Jie Liu, Xin Zhang, Jun Tu, Xianjia Ning, Qing Yang, Jinghua Wang

**Affiliations:** ^1^Department of Cardiology, Tianjin Medical University General Hospital, Tianjin, China; ^2^Department of Neurology, Tianjin Medical University General Hospital, Tianjin, China; ^3^Laboratory of Epidemiology, Tianjin Neurological Institute, Tianjin, China; ^4^Key Laboratory of Post-Neuroinjury Neuro-Repair and Regeneration in Central Nervous System, Tianjin Neurological Institute, Ministry of Education and Tianjin City, Tianjin, China; ^5^Department of Neurosurgery, Tianjin Medical University General Hospital, Tianjin, China; ^6^Center of Clinical Epidemiology & Evidence-Based Medicine, Tianjin Jizhou People's Hospital, Tianjin, China

**Keywords:** carotid atherosclerosis, intima-media thickness, metabolic syndrome, components, epidemiology

## Abstract

**Background:** We aimed to evaluate the relationship between metabolic syndrome (MetS) including its components and carotid intima media thickness (CIMT) in a low-income Chinese population aged ≥45 years.

**Methods:** The participants underwent a general health screening and B-mode carotid ultrasonography that measured CIMT. The diagnosis of MetS and its components was based on the modified International Diabetes Federation Criteria for the Asian Population. The univariate and multivariable linear regression analyses were used to evaluate the relationship between MetS and CIMT.

**Results:** A total of 3,583 participants (mean age, 60 years) was included in the analyses (41.4% male and 58.6% female); more than 50% of the participants were diagnosed with MetS. In the multivariable linear regression analysis, the mean CIMT was 0.009 mm greater in the participants with MetS than in those without MetS (β = 0.009; 95% *CI*, 0.003–0.014; *P* < 0.05). Moreover, a high number of MetS components was associated with greater CIMT values; for example, CIMT increased by 0.007 and 0.015 mm for the individuals diagnosed with 3–4 and 5 MetS components, respectively. Among the MetS components, elevated blood pressure (β = 0.022; 95% *CI*, 0.015–0.029; *P* < 0.001) and abdominal obesity (β = 0.008; 95% *CI*, 0.001–0.015; *P* < 0.001) were positively correlated with CIMT. However, the increased triglyceride levels were negatively associated with CIMT (β = −0.008; 95% *CI*: −0.015 to −0.002; *P* = 0.012), especially among the elderly population.

**Conclusions:** The risk of carotid atherosclerosis increased in the presence of multiple MetS components in a low-income, middle-aged, and elderly population. Accordingly, more detailed management strategies are essential for the early prevention and intervention of atherosclerosis in this low-income population with MetS, in China.

## Introduction

The tremendous burden of cardiovascular diseases (CVDs) (such as stroke) has become a major global public health problem. The CVDs and strokes are the leading causes of death not only in the developed world but also in the underdeveloped countries (1). These diseases are responsible for an estimated 12.1 million deaths in 1990, reaching 18.6 million in 2019, and the number of cardiovascular-related deaths is increasing rapidly (1). A major cause of CVDs and stroke is atherosclerosis (2). Accordingly, the early recognition and intervention of the progress of atherosclerosis are of great significance for reducing the burden of CVDs and stroke in the general population. The ultrasound measurements of the carotid intima-media thickness (CIMT) provide a valuable index of early atherosclerosis, independent of the traditional risk factors, and the CIMT is considered useful for predicting the cardiovascular events and ischemic strokes (3).

Metabolic syndrome (MetS) plays an essential role in the atherosclerotic process as it comprises a cluster of interrelated cardiometabolic risk factors that can increase the risk of atherogenic damage disorder (4). The individuals with MetS were more likely to have new plaques (5, 6), increased CIMT (5, 7, 8), and restenosis after carotid endarterectomy or stenting compared with the non-affected individuals (9). In addition, when larger numbers of MetS components were present, the risk of carotid atherosclerosis also increased. However, most of the previous studies were conducted in developed countries or in higher income, urban areas in China (7, 10–12); conversely, low-income populations have not been well-studied, particularly among individuals aged ≥ 45 years.

Therefore, we performed a population-based, cross-sectional study to evaluate the relationship between MetS and CIMT in a low-income population, aged ≥ 45 years, in China.

## Methods

### Participants and Study Design

This population-based, cross-sectional survey was conducted in the rural areas of Tianjin, China, between April 2014 and January 2015. The study was based on a subset of the population involved in the previously described Tianjin Brain Study (13). Briefly, the Tianjin Brain Study is a population-based stroke surveillance project that includes 14,251 participants from the 18 administrative villages in rural Tianjin, China. The low-income farmers account for approximately 95% of the population, with a per capita disposable income of <1,600 USD, in 2014 (14). The stroke burden in those rural areas was particularly severe among middle-aged adults, with an upward trend in the incidence of first-ever strokes observed between 1992 and 2012 (15).

The current study recruited all the local permanent residents aged ≥ 45 years using the clustering sampling method. Those individuals with histories of coronary heart disease or stroke were excluded because they might have received multiple interventions, such as lifestyle changes or surgical treatments. A total of 5,380 permanent residents were eligible for inclusion in 2014.

All the participants in this study underwent a health screening that included physical examinations, biochemical tests, and carotid ultrasonography.

### Information Collection

The collected sociodemographic and clinical characteristics data included participant name, sex, age, educational level, lifestyle, history of diabetes and hypertension, history of hyperlipidemia, and current use of antihypertensive, antidiabetic, or lipid-lowering agents. The well-trained epidemiology researchers collected these data using face-to-face interviews that were based on a pre-designed standardized questionnaire. The participants were categorized into three age groups: 45–54, 55–64, and ≥ 65 years. Furthermore, the participants were categorized into three groups based on their education level, according to the length of formal education: illiterate (no formal education), primary school (1–6 years), or middle school and above (>6 years). Lifestyle information included smoking status (never, former, or current smoker) and drinking status (never, former, or current drinker).

### Physical Examination and Biochemical Tests

In addition, each participant received a detailed physical examination, such as weight, height, waist circumference (WC), and blood pressure (BP). Body mass index (BMI) was calculated as weight (kg) divided by the square of height in meters (m^2^); the participants were categorized as underweight (BMI <18.50 kg/m^2^), normal (18.50 kg/m^2^ ≤ BMI <24.00 kg/m^2^), overweight (24.00 kg/m^2^ ≤ BMI <28.00 kg/m^2^), or obese (BMI ≥ 28.00 kg/m^2^), according to the Chinese-specific criteria (16). BP was measured with the participant in the sitting position and was calculated using the average of two measurements, at 1-min intervals, after 5 min of rest. To minimize white-coat hypertension, the researchers performed the BP measurements in a quiet room using the standard method described by the American Hypertension Association.

The fasting venous blood samples were collected and sent to the Ji County People's Hospital, within 2 h of collection, for routine examinations; the fasting plasma glucose (FPG) measurements and lipid profiling were performed to determine the levels of total cholesterol (TC), triglyceride (TG), high-density lipoprotein cholesterol (HDL-C), and low-density lipoprotein cholesterol (LDL-C).

### Ultrasonography Measurements

All ultrasound examinations and measurements were performed by one trained technician, blinded to the details of the participants. The participants were examined while they were in a supine position using B-mode ultrasonography (Terason 3000, Burlington, MA, USA) with a 5–12 MHz linear array transducer. The bilateral, extracranial carotid artery trees (including the common carotid artery, carotid sinus, and internal and external carotid arteries) were screened for CIMT. CIMT of the near and far walls of the common carotid artery was measured on the left and the right sides. The maximum, minimum, and average CIMT values for each side were obtained. The average CIMT was calculated using the means for the sum of the CIMTs on both the left and right sides. The images were obtained and digitally stored according to a standard protocol (17). All the scans were recorded on Vascular Research Tools 6 (MIA, LLC, IA, USA) for subsequent off-line analysis. The inter-observer and intra-observer correlation coefficients ranged from 0.88 to 0.94 and 0.80 to 0.95 for both sides of the CIMT measurement, respectively.

### Criteria for Metabolic Syndrome

Metabolic syndrome was defined as the presence of three or more of the following according to the modified International Diabetes Federation criteria for the Asian Population, published in 2009(18): (1) abdominal obesity: WC ≥ 90 cm for men and ≥ 80 cm for women; (2) increased TG levels: TG ≥ 1.70 mmol/L (150 mg/dl) or using medications to treat the increased levels of TG; (3) reduced HDL-C: HDL-C <1.0 mmol/L (40 mg/dl) for men and <1.3 mmol/L (50 mg/dL) for women, or on drug treatment for low HDL-C; (4) elevated BP: systolic BP ≥ 130 mmHg and/or diastolic BP ≥ 85 mmHg, and/or use of antihypertensive medication; (5) elevated FPG: FPG ≥ 5.6 mmol/L (100 mg/dl) and/or taking medication(s) for diabetes.

The five criteria described above were designated as the MetS components. The participants were divided into three groups according to the number of MetS components present upon enrollment: 0–2, 3–4, and 5 components.

### Statistical Analysis

The continuous variables (age, BMI, education, WC, systolic BP, diastolic BP, FPG, TC, TG, HDL-C, LDL-C, and CIMT) are presented as means (SDs) or medians (interquartile ranges [IQRs]). The comparisons between the groups were performed using Student' s *t*-test, the Mann–Whitney test, or the Kruskal–Wallis test if appropriate. The categorical variables (sex, age group, education level, BMI group, smoking status, alcohol consumption, and presence of MetS and the number of its components) are presented as the numbers and frequencies. Fisher' s exact test was used for comparisons among the categorical variables. The univariate and multivariable linear regression analyses were applied to determine the association among the CIMT and MetS, MetS components, and the number of MetS components; the results are presented as β-coefficients and 95% *CI*s. The independent variables comprised variables for which *P* < 0.05 in the univariate linear regression analysis. The statistical significance was defined as a two-tailed value of *P* < 0.05. All the statistical analyses were performed using the SPSS software (version 22.0; SPSS, Chicago, IL, USA).

## Results

**Figure 1:**
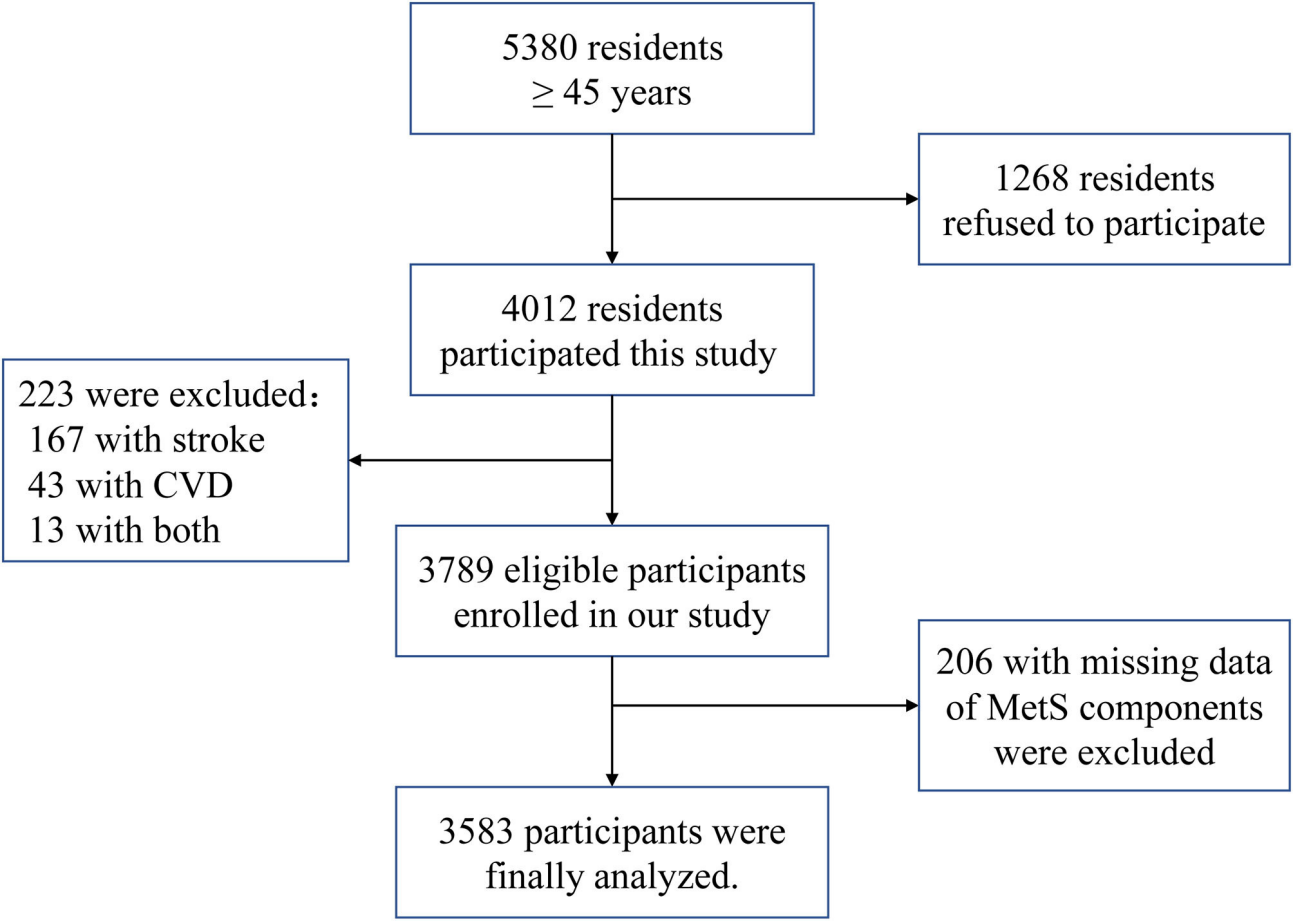
The flow chart of participants selection.

Among the 5,380 qualified residents, we initially excluded 1,268 residents who did not provide informed consent and an additional 223 participants with histories of coronary heart disease or stroke. Additionally, we excluded 206 participants with missing data regarding the MetS components. Therefore, the final sample consisted of 3,583 participants (Figure 1).

### Baseline Characteristics

Of the 3,583 participants (41.4% men; mean age 60 years) included in the analysis, 54.5% were diagnosed with MetS. More than 60% of the participants had received a primary level of education or less, with a median education level of 6 years in both the MetS and non-MetS groups. A quarter of the participants were current or ever smokers, and 15.7% consumed alcohol. Moreover, 42.2% of participants were overweight, and nearly 25% were obese. Furthermore, 29.0% of the participants were taking at least one antihypertensive drug, whereas only 0.5% were taking a lipid-lowering agent. The average values for BMI (25.53 kg/m^2^), WC (89.15 cm), FBG (5.92 mmol/L), TC (4.86 mmol/L), TG (1.75 mmol/L), HDL-C (1.46 mmol/L), LDL-C (2.70 mmol/L), systolic BP (146.53 mmHg), diastolic BP (86.84 mmHg), and CIMT (0.57 mm) were determined (Table 1).

**Table 1:** Characteristics of study participants.

**Category**	**Total**	**MetS**	**Non-MetS**	***P-*value**
Total	3,583 (100)	1,954 (54.5)	1,629 (45.5)	
Sex, *n* (%)				<0.001
Men	1,482 (41.4)	627 (32.1)	855 (52.5)	
Women	2,101 (58.6)	1,327 (67.9)	774 (47.5)	
Age, means (SD), years	60.06 (9.52)	60.35 (9.31)	59.71 (9.77)	0.045
Age group, *n* (%)				0.033
45~54 years	1,120 (31.3)	577 (29.5)	543 (33.3)	
55~64 years	1,410 (39.4)	777 (39.8)	633 (38.9)	
≥ 65 years	1,053 (29.4)	600 (30.7)	453 (27.8)	
Education, median (IQR), years	6 (6)	6 (6)	6 (6)	<0.001
Education level, *n* (%)				<0.001
0 years	632 (17.6)	391 (20.0)	241 (14.8)	
1~6 years	1,604 (44.8)	863 (44.2)	741 (45.5)	
> 6 years	1,347 (37.6)	700 (35.8)	647 (39.7)	
BMI group, *n* (%)				<0.001
Low weight	65 (1.8)	6 (0.3)	59 (3.6)	
Normal	1,177 (32.8)	338 (17.3)	839 (51.5)	
Overweight	1,511 (42.2)	940 (48.1)	571 (35.1)	
Obesity	830 (23.2)	670 (34.3)	160 (9.8)	
Smoking status, *n* (%)				<0.001
Never smoking	2,688 (75.0)	1,575 (80.6)	1,113 (68.3)	
Current or ever smoking	895 (25.0)	379 (19.4)	516 (31.7)	
Alcohol consumption, *n* (%)				<0.001
Never drinking	3,019 (84.3)	1,703 (87.2)	1,316 (80.8)	
Current or ever drinking	564 (15.7)	251 (12.8)	313 (19.2)	
**Medication history**, ***n*** **(%)**
Anti-hypertensive	1,038 (29.0)	729 (37.3)	309 (19.0)	<0.001
Anti-diabetes	196 (5.5)	170 (8.7)	26 (1.6)	<0.001
Lipid lowering agent	19 (0.5)	18 (0.9)	1 (0.1)	<0.001
BMI, means (SD), kg/m^2^	25.53 (3.69)	26.93 (3.39)	23.86 (3.32)	<0.001
WC, means (SD), cm	89.15 (9.08)	92.86 (7.91)	84.69 (8.37)	<0.001
FBG, means (SD), mmol/L	5.92 (1.57)	6.35 (1.77)	5.39 (1.06)	<0.001
TC, means (SD), mmol/L	4.86 (1.08)	5.03 (1.13)	4.66 (0.97)	<0.001
TG, means (SD), mmol/L	1.75 (1.22)	2.21 (1.39)	1.19 (0.60)	<0.001
HDL-C, means (SD), mmol/L	1.46 (0.46)	1.33 (0.43)	1.63 (0.44)	<0.001
LDL-C, means (SD), mmol/L	2.70 (1.26)	2.81 (1.35)	2.56 (1.12)	<0.001
SBP, means (SD), mmHg	146.53 (22.12)	151.35 (20.70)	140.75 (22.38)	<0.001
DBP, means (SD), mmHg	86.84 (11.43)	89.16 (11.02)	84.05 (11.29)	<0.001
**MetS components**, ***n*** **(%)**
Elevated BP	2,919 (81.5)	1,813 (92.8)	1,106 (67.9)	<0.001
Elevated FBG	1,804 (51.1)	1,423 (72.8)	381 (24.2)	<0.001
Reduced HDL-C	1,012 (28.7)	903 (46.2)	109 (6.9)	<0.001
Elevated TG	1,323 (37.5)	1,189 (60.8)	134 (8.5)	<0.001
Abdominal obesity	2,534 (70.9)	1,815 (93.0)	719 (44.3)	<0.001
CIMT, mean (SD), mm	0.57 (0.09)	0.57 (0.08)	0.56 (0.09)	0.055

*MetS, Metabolic syndrome; IQR, interquartile range; SD, standard deviation; SBP, systolic blood pressure; DBP, diastolic blood pressure; BP, blood pressure; BMI, body mass index; WC, Waist circumference; FBG, fasting blood glucose; TC, total cholesterol; TG, triglycerides; HDL-C, high density lipoprotein cholesterol; LDL-C, low density lipoprotein cholesterol*.

The total prevalence of MetS, in this study, was 54.5%. Among the MetS components, the most prevalent components were increased BP (81.5%) and abdominal obesity (70.9%); reduced HDL-C (28.7%) was the least prevalent of the five MetS components. The individuals with 0, 1, 2, 3, 4, and 5 MetS components accounted for 3.3, 16.0, 26.2, 27.3, 18.8, and 8.5%, respectively, of the participants (Table 2).

**Table 2:** Characteristics of study participants stratified by MetS components.

**Category**	**0 components**	**One component**	**Two components**	**Three components**	**Four components**	**Five components**	***P-*value**
Total	118 (3.3)	573 (16.0)	938 (26.2)	977 (27.3)	673 (18.8)	304 (8.5)	
Sex, *n* (%)							<0.001
Men	70 (59.3)	330 (57.6)	455 (48.5)	352 (36.0)	199 (29.6)	76 (25.0)	
Women	48 (40.7)	243 (42.4)	483 (51.5)	625 (54.0)	474 (70.4)	228 (75.0)	
Age, means (SD), years	57.70 (9.73)	59.34 (9.63)	60.18 (9.82)	60.79 (9.83)	59.80 (8.70)	60.14 (8.82)	0.005
Age group, *n* (%)							0.030
45~54 years	48 (40.7)	202 (35.3)	293 (31.2)	289 (29.6)	204 (30.3)	84 (27.6)	
55~64 years	42 (35.6)	215 (37.5)	376 (40.1)	364 (37.3)	280 (41.6)	133 (43.8)	
≥ 65 years	28 (23.7)	156 (27.2)	269 (28.7)	324 (33.2)	189 (28.1)	87 (28.6)	
Education, median (IQR), years	6.5 (5)	6.0 (5.0)	6.0 (6.0)	6.0 (7.0)	6.0 (7.0)	6.0 (6.0)	<0.001
Education level, *n* (%)							<0.001
0 years	15 (12.7)	71 (12.4)	155 (16.5)	197 (20.2)	133 (19.8)	61 (20.1)	
1~6 years	44 (37.3)	264 (46.1)	433 (46.2)	422 (43.2)	302 (44.9)	139 (45.7)	
> 6 years	59 (50.0)	238 (41.5)	350 (37.3)	358 (36.6)	238 (35.4)	104 (34.2)	
BMI group, *n* (%)							<0.001
Low weight	11 (9.3)	33 (5.8)	15 (1.6)	5 (0.5)	1 (0.1)	0 (0)	
Normal	95 (80.5)	344 (60.0)	400 (42.6)	218 (22.3)	98 (14.6)	22 (7.2)	
Overweight	12 (10.2)	175 (30.5)	384 (40.9)	495 (50.7)	298 (44.3)	147 (48.4)	
Obesity	0 (0)	21 (3.7)	139 (14.8)	259 (26.5)	276 (41.0)	135 (44.4)	
Smoking status, *n* (%)							<0.001
Never smoking	74 (62.7)	366 (63.9)	673 (71.7)	775 (79.3)	550 (81.7)	250 (82.2)	
Current or ever smoking	44 (37.3)	207 (36.1)	265 (28.3)	202 (20.7)	123 (18.3)	54 (17.8)	
Alcohol consumption, *n* (%)							<0.001
Never drinking	97 (82.2)	455 (79.4)	764 (84.7)	828 (84.7)	599 (89.0)	276 (90.8)	
Current or ever drinking	21 (17.8)	118 (20.6)	174 (18.6)	149 (15.3)	74 (11.0)	28 (9.2)	
**Medication history**, ***n*** **(%)**
Anti-hypertensive	0 (0)	76 (13.3)	233 (24.8)	333 (34.1)	250 (37.1)	146 (48.0)	<0.001
Anti-diabetes	0 (0)	7 (1.2)	19 (2.0)	70 (7.2)	65 (9.7)	35 (11.5)	<0.001
Lipid lowering agent	0 (0)	0 (0)	1 (0)	6 (0.6)	9 (1.3)	3 (1.0)	<0.001
BMI, means (SD), kg/m^2^	1.01 (0.43)	1.32 (0.64)	1.69 (0.74)	2.03 (0.71)	2.26 (0.70)	2.37 (0.62)	<0.001
WC, means (SD), cm	77.78 (6.05)	81.85 (7.26)	87.29 (8.20)	91.46 (7.78)	93.85 (7.77)	95.16 (7.81)	<0.001
FBG, means (SD), mmol/L	4.97 (0.36)	5.20 (0.89)	5.55 (1.17)	6.02 (1.32)	6.56 (2.06)	6.92 (2.09)	<0.001
TC, means (SD), mmol/L	4.67 (1.01)	4.60 (0.92)	4.69 (0.99)	4.96 (1.06)	5.14 (1.23)	5.02 (1.08)	<0.001
TG, means (SD), mmol/L	0.96 (0.34)	1.08 (0.45)	1.28 (0.69)	1.70 (0.96)	2.41 (1.25)	3.40 (1.95)	<0.001
HDL-C, means (SD), mmol/L	1.75 (0.37)	1.67 (0.42)	1.59 (0.46)	1.47 (0.47)	1.26 (0.35)	1.03 (0.17)	<0.001
LDL-C, means (SD), mmol/L	2.47 (1.03)	2.47 (1.15)	2.62 (1.11)	2.79 (1.17)	2.89 (1.41)	2.69 (1.72)	<0.001
SBP, means (SD), mmHg	117.23 (8.10)	137.29 (22.17)	145.83 (21.42)	149.98 (20.91)	151.68 (20.86)	155.03 (19.24)	<0.001
DBP, means (SD), mmHg	73.80 (6.46)	82.48 (11.00)	86.30 (11.07)	87.95 (10.80)	89.88 (11.26)	91.45 (10.69)	<0.001
CIMT, mean (SD), mm	0.54 (0.08)	0.56 (0.09)	0.57 (0.09)	0.57 (0.09)	0.56 (0.07)	0.57 (0.09)	0.001

*MetS, Metabolic syndrome; IQR, interquartile range; SD, standard deviation; SBP, systolic blood pressure; DBP, diastolic blood pressure; BP, blood pressure; BMI, body mass index; WC, Waist circumference; FBG, fasting blood glucose; TC, total cholesterol; TG, triglycerides; HDL-C, high density lipoprotein cholesterol; LDL-C, low density lipoprotein cholesterol*.

### Relationship Between MetS and CIMT in the Univariable Linear Regression Analysis

Table 3 shows the results of the univariable analysis to estimate the relationship between MetS and CIMT. The participants with MetS exhibited a positive association between MetS and CIMT. Significant differences were found among the four MetS components: increased BP, increased FBG, reduced HDL-C, and increased TG (all *P* < 0.05). The TC and LDL-C levels were positively associated with increased CIMT (β = 0.003 and 0.007, respectively; both *P* < 0.05). Moreover, sex, age, education, smoking, and alcohol consumption were associated with the CIMT values.

**Table 3:** Relationship between MetS and CIMT in the univariate liner regression analysis.

**Risk factors**	**β (95%CI)**	***P*-value**
MetS	0.006 (0, 0.011)	0.053
**Num. of components of MetS**
0–2	Ref.	
3–4	0.005 (−0.001, 0.011)	0.113
5	0.010 (−0.001, 0.021)	0.064
**Single components of MetS**
Elevated BP	0.036 (0.028, 0.043)	<0.001
Elevated FBG	0.013 (0.007, 0.018)	<0.001
Reduced HDL-C	−0.004 (−0.010, 0.003)	0.003
Elevated TG	−0.008 (−0.014, −0.002)	0.012
Abdominal obesity	−0.004 (−0.010, 0.002)	0.213
**Sex**		<0.001
Women	Ref.	
Men	0.028 (0.022, 0.034)	
**Age group**
45~54 years	Ref.	
55~64 years	0.037 (0.030, 0.043)	<0.001
≥ 65 years	0.062 (0.054, 0.069)	<0.001
**Education group**
0 years	Ref.	
1~ 6 years	−0.010 (−0.018, −0.002)	0.015
> 6 years	−0.033 (−0.041, −0.025)	<0.001
**BMI group**
Low weight	0.025 (0.003, 0.047)	0.026
Normal	Ref.	0.602
Overweight	0.002 (−0.005, 0.008)	0.103
Obesity	0.006 (−0.001, 0.014)	
**Smoking status**
Never smoking	Ref.	
Current or ever smoking	0.020 (0.013, 0.027)	<0.001
**Alcohol consumption**
Never drinking	Ref.	
Current or ever drinking	0.021 (0.013, 0.029)	<0.001
TC	0.003 (0.001, 0.006)	0.014
LDL–C	0.007 (0.004, 0.009)	<0.001

*BMI, body mass index; MetS, Metabolic syndrome; FBG, fasting blood glucose; BP, blood pressure; TC, total cholesterol; TG, triglycerides; HDL–C, high density lipoprotein cholesterol; LDL–C, low density lipoprotein cholesterol*.

### Relationship Between MetS and CIMT in Multivariable Linear Regression Analysis

As shown in Table 4, after adjusting for age, sex, education, smoking, alcohol consumption, TC, and LDL-C, the mean participant CIMT increased 0.009 mm in the participants with MetS compared with those without MetS (β = 0.009; 95% *CI*, 0.003–0.014; *P* = 0.003). Relative to the number of MetS components present in the participants, the CIMT values increased by 0.007 and 0.015 mm in the participants with 3–4 and 5 components, respectively, compared with the participants with <3 components. Among the MetS components, the increased BP and abdominal obesity were positively correlated with CIMT. CIMT increased by 0.022 mm (β = 0.022; 95% *CI*, 0.015–0.029; *P* < 0.001) in the participants with increased BP and 0.008 mm (β = 0.008; 95% *CI*, 0.001–0.015; *P* < 0.001) in those with abdominal obesity. However, the increased triglycerides levels were negatively associated with CIMT value, with the mean CIMT decreasing by 0.008 mm (β = −0.008; 95% *CI*, −0.015 to −0.002; *P* = 0.012) compared with those with normal triglyceride level. The subgroup analysis revealed that the association between raised TG and CIMT was more pronounced among the participants ≥ 60 years (β = −0.014; 95% *CI*, −0.024 to −0.004), compared with those with <60 years (β = −0.002; 95% *CI*, −0.010 to 0.006) at baseline (*P* interaction = 0.002) (Supplementary Table).

**Table 4:** Multivariable liner regression analysis of relationship between MetS, MetS components, and number of MetS components and CIMT.

**Risk factors**	**β (95%CI)**	***P*-value**
MetS	0.009 (0.003, 0.014)	0.003
**Num. of MetS components**
0–2	Ref.	
3–4	0.007 (0.001, 0.013)	0.015
5	0.015 (0.005, 0.026)	0.004
**MetS components**
Elevated BP	0.022 (0.015, 0.029)	<0.001
Elevated FBG	0.003 (−0.003, 0.008)	0.326
Reduced HDL–C	0.005 (−0.002, 0.012)	0.128
Elevated TG	−0.008 (−0.015, −0.002)	0.012
Abdominal obesity	0.008 (0.001, 0.015)	0.022

*MetS, Metabolic syndrome; FBG, fasting blood glucose; BP, blood pressure; TG, triglycerides; HDL-C, high density lipoprotein cholesterol. Multivariate analysis in MetS, Num. of MetS components and MetS components were adjusted by age, sex, education, smoking, alcohol consumption, TC and LDL-C*.

## Discussion

In the present study, we demonstrated a high prevalence (54.5%) of MetS among the low-income residents (aged ≥ 45 years) in an area of China. Further, the individuals with MetS were more likely to have the increased CIMT values than the non-affected individuals. Among the MetS components, increased BP and abdominal obesity were positively correlated with CIMT, whereas increased TG levels were negatively associated with CIMT. Moreover, CIMT increased significantly when more components of MetS were present. Our results provide new information and evidence for the relationship between the MetS, including its components, and CIMT in a low-income, rural, middle-aged, and elderly population with a high incidence of strokes.

The previous cross-sectional studies demonstrated that the individuals with MetS have increased CIMTs (7, 19). In addition, an increasing trend in the CIMT values was observed with an increasing number of MetS components in both genders (20, 21). Moreover, a cross-sectional study from northern China found that the average CIMT was higher in the participants with MetS (β = 0.020; 95% *CI*, 0.014–0.027; *P* < 0.001) and that CIMT increased by 0.022, 0.042, and 0.035 mm for those with 3, 4, and 5 MetS components, respectively (7). Another study, from Hong Kong, reported results consistent with those in the present study, showing that the MetS components were independently related to CIMT and that the risk of increased CIMT was significantly higher when the participants had increased numbers of MetS components, compared with those without MetS components (11). Similar to these previous studies, the current results suggest that the mean CIMT is higher in the participants with MetS than in those without MetS, and that there is a greater risk of increased CIMT when an individual has more MetS components present.

Individual abnormalities in the MetS components are traditional risk factors for CVD and stroke. For example, hypertension is a major risk factor for the development of atherosclerosis (22). Citing more than 13 years of a follow-up study, the Tromsø study, from Norway, reported that MetS, especially the hypertension component, was associated with increased CIMT (6). Additionally, a cross-sectional study of 8,144 apparently healthy Japanese individuals reported that hypertension was the most common MetS component and the greatest contributor to carotid arteriosclerosis (12). The present results are also in accordance with these previous studies, showing that, among all the MetS components, increased levels of BP contributed the greatest risk for increased CIMT. Compared with the participants without MetS, the mean CIMT for individuals with higher BP increased by 0.022 mm; another component, abdominal obesity, was associated with a 0.008-mm increase in CIMT. These data re-emphasize the need for clinicians to pay more attention to the impact of lowering BP for reducing the risk of both MetS and carotid arteriosclerosis.

Obesity involves a chronic inflammatory process characterized by an increase in various proinflammatory biomarkers, which may mediate all the stages of atherogenesis, such as the initiation, acceleration, and progression of atherosclerotic lesions (23). The Brazilian Longitudinal Study of Adult Health reported that, based on multivariate linear analysis, with each 1-cm increase in WC, CIMT increased 0.08 mm (β = 0.08; 95% *CI*, 0.04–0.11; *P* < 0.0001) (24). Another study conducted in Shanghai, China showed that WC was an independent risk factor for increased CIMT, with WC ≥ 80, 81–84, and ≥ 85 cm exhibiting odds ratios (*OR*s) of 1.632, 1.501, and 1.878, respectively (25). Moreover, a meta-analysis found that obesity has a long-term effect on CIMT, with childhood obesity significantly (2.5-fold) increasing the risk of a large CIMT in adulthood (*OR* = 3.5; 95% *CI*, 1.1–11.1; *P* = 0.034) (26). In the present study, WC was measured as an indicator of abdominal obesity, and we found that WC was positively associated with carotid atherosclerosis. The CIMT value in the individuals with abdominal obesity was 0.008-mm greater than in the individuals without abdominal obesity.

A 12-year longitudinal study revealed that abnormal FPG levels were also a risk factor for atherosclerosis (27). The Jackson Heart Study of 4,303 community-dwelling blacks revealed that each 10-mg/dl increase in FPG was associated with higher odds of subclinical CVD, such as left ventricular hypertrophy, coronary artery calcification, increased CIMT (all *P* < 0.005) after being adjusted for traditional CVD risk factors (28). However, a 10-year follow-up of the Hoorn Study showed no significant correlation between FPG and an increased risk of non-fatal CVD (29). Additionally, some studies have reported that the low HDL-C levels are protective against the high CIMT values (30, 31). However, among the patients with MetS, no association was found between the reduced HDL-C levels and carotid arteriosclerosis, after age stratification (12). In contrast, the present results showed that the increased FPG levels and reduced HDL-C levels had no significant bearing on CIMT. Nonetheless, since a cross-sectional study is based on data collected at a certain time point, the present data may not be able to verify a correlation between the HDL-C or FPG levels and CIMT.

The relationship between the TG levels and carotid atherosclerosis remains controversial. A previous study reported that the high TG levels were a risk factor for atherosclerosis (hazard ratio = 1.003; 95% *CI*, 1.00–1.006; *P* = 0.027) (32). However, a Mendelian randomization analysis reported no causal association between the TG levels and CIMT (33). Furthermore, another study of 6,142 Chinese individuals reported that hypertriglyceridemia was associated with a 20% reduction in risk of carotid atherosclerosis, but only in men (10). The present results showed that the TG levels protected against increased CIMT values; the CIMT value in individuals with the increased TG level decreased by 0.008 mm compared with the participants with the normal TG levels. The underlying reason for the negative correlation between elevated TG and CIMT remains unclear. However, the result in our study may be partially explained by the different distribution of TG and CIMT by age. The previous studies have shown that the age-related TG distribution was gradually declined after midlife due to the reduction of lipids absorption, hormonal changes, and decline of health status (10, 34). On the contrary, the CIMT was increasing with age (35, 36). The subgroup analysis in our study showed that the negative association between raised TG and CIMT was more pronounced among the participants ≥ 60 years. With the increasing of age, TG and CIMT show the opposite trend, which leads to higher TGs having the protect effect in CIMT, and the effect is more obvious in elderly patients. However, it still needs more studies to explore the relationship between TG and CIMT in different populations.

Atherosclerosis is the underlying process of the majority of CVDs and CVD-associated mortality. Non-invasive ultrasonographic assessment of CIMT is suitable for evaluating the early burden of atherosclerosis and predicting future CVD risks. Moreover, the burden of stroke among the low-income populations in rural areas remains severe; previous research illustrated that the incidence of stroke in one such area increased by 6.5%, annually, between 1992 and 2012 (15). MetS has been highlighted as a major, global socioeconomic problem owing to the high prevalence of MetS in aging societies and it is being significantly associated with adverse cardiovascular events. Therefore, the relationship between MetS and CIMT may indicate that MetS is associated with the initiation of the atherosclerotic process. This observation is of great significance for the early recognition and prevention of atherosclerosis progression and more detailed management strategies in individuals with MetS.

### Limitations

The present study has several limitations. First, as observed in many other cross-sectional studies, the present results cannot validate the causal links between MetS and CIMT. Further cohort studies, with follow-up data, are needed to verify this possibility. Second, this is a population-based study from a single township in a low-income, rural population in Tianjin, China; therefore, this study is not representative of the entire population. Third, the study population included only participants aged ≥ 45 years, meaning that these findings cannot be generalized to broader age groups. However, with the advancing age of many societies and the poor lifestyles in those societies, there will likely be an increase in the age-specific prevalence of MetS among older adults (37). Hence, exploring MetS in individuals aged ≥ 45 years is both necessary and meaningful. Finally, the research failed to capture some potential confounding factors, such as dietary habits, which may affect the relationship between the raised TG level and CIMT. Despite part of the explanation being the different distribution of TG and CIMT by age, the underlying mechanisms remain unclear. Subsequent studies are needed to provide additional explanations.

## Conclusion

In conclusion, our study showed that the individuals with MetS, particularly those with greater numbers of MetS components, had a substantial risk of having elevated CIMT values. The increased levels of BP and abdominal obesity had a positive correlation with CIMT. While the increased TG levels were negatively associated with CIMT, especially among the elderly population. These findings suggested that detailed and strict MetS management strategies should be encouraged to identify and intervene in the atherosclerosis process among the low-income populations in China.

## Data Availability Statement

The raw data supporting the conclusions of this article will be made available by the authors, without undue reservation.

## Ethics Statement

The studies involving human participants were reviewed and approved by the Ethics Committee of Tianjin Medical University General Hospital. The patients/participants provided their written informed consent to participate in this study.

## Author Contributions

XN, JW, and QinY were involved in the conception and design and data interpretation for this article. QiaY, QL, DG, HW, JL, XZ, and JT were involved in data collection, case diagnosis, and confirmation for this article. QiaY, QL, and DG were involved in manuscript drafting. JW was involved in data analysis for this article. XN, JW, and QinY were involved the critical review of this article. All authors contributed to the article and approved the submitted version.

## Conflict of Interest

The authors declare that the research was conducted in the absence of any commercial or financial relationships that could be construed as a potential conflict of interest. The reviewers XY and YG declared a shared affiliation with all of the authors to the handling editor at the time of the review.

## Publisher's Note

All claims expressed in this article are solely those of the authors and do not necessarily represent those of their affiliated organizations, or those of the publisher, the editors and the reviewers. Any product that may be evaluated in this article, or claim that may be made by its manufacturer, is not guaranteed or endorsed by the publisher.
