# Supplementary material for: Association of Carotid Intima Media Thickness With Metabolic Syndrome Among Low-Income Middle-Aged and Elderly Chinese: A Population-Based Cross-Sectional Study
Source: Front Cardiovasc Med. 2021 Nov 19;8:669245. doi: 10.3389/fcvm.2021.669245 (PMC8639590; doi:10.3389/fcvm.2021.669245)
Supplement: Supplementary file 1 [file Table_1.DOCX]

Supplementary Table. Multivariate liner regression analysis of relationship between raised TG and CIMT stratified by risk factors

| Risk factors | β (95%CI) | *P* interaction |
| --- | --- | --- |
| Age group |  | 0.002 |
| <60 years | -0.002 (−0.010, 0.006) |  |
| ≥60 years | -0.014 (−0.024, -0.004) |  |
| Sex |  | 0.932 |
| Men | -0.010 (-0.021, 0.002) |  |
| Women | -0.007 (-0.015, 0.001) |  |
| Education group |  | 0.607 |
| Illiteracy | -0.009 (-0.026, 0.009) |  |
| Non-illiteracy | -0.008 (-0.015, -0.001) |  |
| Smoking |  | 0.939 |
| Never smoking | -0.007 (-0.014, 0.001) |  |
| Current or ever smoking | -0.012 (-0.027, 0.002) |  |
| Drinking |  | 0.790 |
| Never drinking | -0.007 (-0.014, -0.001) |  |
| Current or ever drinking | -0.014 (-0.033, 0.006) |  |
